# Hybridization *vs.* Bond Stretching Isomerism in Ru(II) Cyclometalated Complexes of 2-Phenylpyridine

**DOI:** 10.3390/molecules17010034

**Published:** 2011-12-22

**Authors:** Bertha Molina, Larissa Alexandrova, Ronan LeLagadec, Luis E. Sansores, David Rios-Jara, Francisco Espinosa-Magaña, Roberto Salcedo

**Affiliations:** 1 Facultad de Ciencias, Universidad Nacional Autónoma de México, Circuito Exterior s/n, Ciudad Universitaria, Coyoacán, Apdo. Post. 70-646, 04510 México D.F., Mexico; 2 Instituto de Investigaciones en Materiales, Universidad Nacional Autónoma de México, Circuito Exterior s/n, Ciudad Universitaria, Coyoacán 04510 México D.F., Mexico; 3 Instituto de Química, Universidad Nacional Autónoma de México, Circuito Exterior s/n, Ciudad Universitaria, Coyoacán 04510 México D.F., Mexico; 4 Instituto Potosino de Investigación Científica y Tecnológica A.C., Camino a la presa de San José 2055, San Luis Potosí, SLP 78216, Mexico; 5 Centro de Investigaciones en Materiales Avanzados, SC, Miguel Angel de Cervantes 120, Chihuahua, Chi. 31109, Mexico

**Keywords:** hybridization isomerism, bond stretch isomerism, Ru organometallic complexes, *trans* effect, EELS spectroscopy, time dependent DFT calculations

## Abstract

The phenomenon of formation of two isomers, yellow and orange, of the cyclometalated Ru(II) complex, [Ru(*o*-C_6_H_4_-py)(MeCN)_4_]^+^, was investigated by EELS spectroscopy and theoretical calculations. Both forms show very similar structures and spectroscopic properties, but slight differences in X-ray data and absorption between them were noted. No double minimum on the potential energy surface was found and thus these two forms cannot be considered as bond stretching isomers. However, the DFT study revealed the change in the hybridization of the carbon in *trans*-position of one of acetonitrile ligands. This effect can be responsible for the difference in colour. The results of the theoretical modelling coincide well with the experimental EELS data.

## 1. Introduction

The concept of “bond stretch isomerism” was first introduced by Hoffmann based on the theoretical simulation of strained organic tricyclic molecules and was shortly followed by theoretical analyses of other systems [1]. The concept implies an existence of two stable conformations, which are different only by the length of one or more chemical bonds [1,2]. However it took more than a decade to find experimental evidence in support of this very rare kind of isomerism. The early attempts to isolate and to characterize bond-stretch isomers were problematic and many of them were considered as crystallographic artifacts [2,3,4]. Even nowadays the number of compounds which demonstrate this isomerism is very limited. Thus, according to Rohmer and Bénard [4], the first stretch isomers that had been isolated and fully characterized by Niecke *et al.* [5] were the biradical 1,3-diphosphacyclobutane-2,4-diyl and its corresponding bicyclic form. Later Bertrand *et al.* [6,7] reported the co-existence in solution of two bond-stretch isomers with *trans*-annular π bonding overlap. The scope of data on the stretching bonds in biradicals has been recently summarized by Breher [8,9] in a very nice review.

Probably the most numerous examples of these isomers have been reported for transition metal complexes. This arises from the large variety of possible structures which may be synthesized for one metal centre using even the same ligands. Many factors such as a small difference between low lying empty molecular orbitals of metal and high lying occupied orbitals of ligand, *trans* effect, crystallization conditions, *etc.*, can all affect the resulting crystal packing.

Thus, structural variability caused by deviation in bond length was found in the dinuclear ruthenium compound, [(Cp*RuCl)_2_(μ-Cl)_2_] [10,11], in trinuclear cobalt [4,12,13,14] and chromium complexes [15]. Recently, two conformations of a dinuclear iron complex differing in valence angles and bond length were reported by Hammann *et al.* [16]. The possibility of stretch isomerism in the trinuclear molybdenum compound [Mo_3_X_12_]^3^^−^ (X = F, Cl, Br, I), was explored by Cavigliasso and Stranger [17]. Additionally, Comba *et al.* [18] described the distortional isomerism observed in copper(I) complexes of 3,7-diazabicyclo[3.3.1] nonane derivatives.

As can be seen, the grand majority of the complexes explored for the bond-stretch isomerism are many nuclear (di- or tri-) compounds and the biggest variation in distance between atoms was always observed in the metal framework. The difference in metal-metal distance varied approximately between more than 1 Å and several tenths of an Å, meanwhile the variations in bond length of ligands or metal-ligand were much smaller. The strict definition of bond-stretch isomerism implies the existence of a double minimum on the potential energy surface of an investigated compound associated with a specific spin multiplicity [2,19]. Therefore there is a very faint border between bond-stretch and spin-state isomerism for metal complexes, particularly in non-mononuclear ones, where variation in metal-metal distance may result in a change in multiplicity. The thorough theoretical analysis together with accurate experimental measurements is required for determination of this isomerism.

Hybridization isomerism is a very rare concept [20,21], there are few examples of this strange phenomenon, whose definition is “isomerism caused by the change in hybridization on one of the atoms in a pair of species”. It has been cited by Collman [20] in NO coordination complexes and for the same reason of the limited cases there are no reviews or deep discussions about it, however the phenomenon plays an important role in the present research.

**Figure 1:**
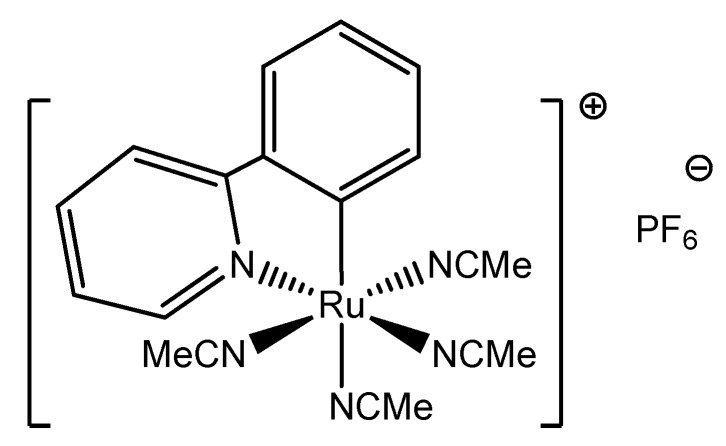
Structure of [Ru(*o*-C_6_H_4_-py)(MeCN)_4_]^+^, the compound under the study.

The cyclometalated ruthenium(II) compound, [Ru(*o*-C_6_H_4_-py)(MeCN)_4_]^+^ (see Figure 1) analyzed in this work was successfully used in ligand exchange reactions because of its labile MeCN ligands. It reacts readily with 1,10-phenanthroline (phen) in MeCN to afford [Ru(*o*-C_6_H_4_-py)(phen)(MeCN)_2_]^+^ but the same reaction with 2,2′-bipyridine (bpy) resulted in a significant colour change from yellow to brownish orange, but without any notable structural changes [22,23]. The new orange compound turned out to be slightly more stable than the yellow one and additionally a small difference between them was also observed by UV-Vis spectroscopy. In the first communication on the subject the presence of two structures was suggested [24], but an explanation for this strange phenomenon was not provided since more studies were deemed necessary. Detailed analysis of the X-ray data revealed slight difference in the several bond lengths between the yellow and orange compounds. In the present article the two structures were characterized by electron energy-loss spectroscopy (EELS) in an attempt to find out more distinguished features between them. EELS is a powerful analytical technique that can give information about composition and optical properties of materials [25], where the difference between the yellow and orange forms is very notable. We also intend to further investigate, by means of DFT calculations, a hypothesis whether these two forms may be considered as bond stretch isomers or are a results of a change in the hybridization.

## 2. Results and Discussion

**Figure 2:**
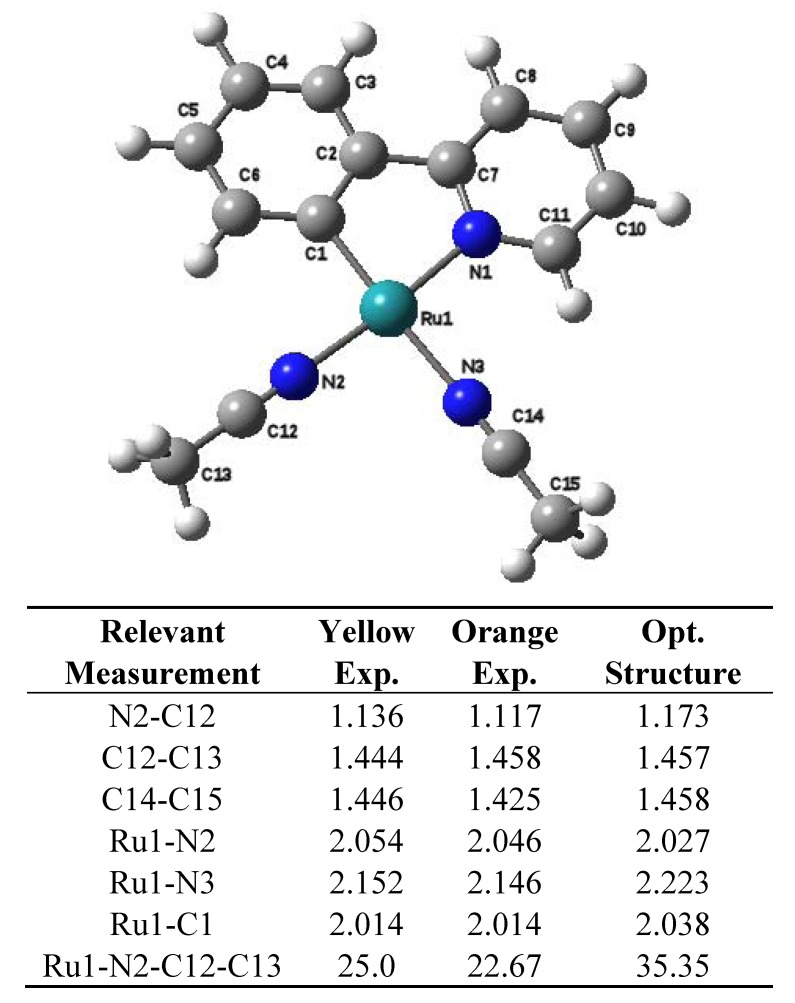
Fragment of the compound in the equatorial plane and relevant distances.

The large similarity of the X-ray data of both isomers of this complex (denoted as yellow and orange) has been established before [24]. However, a more careful examination of the bond lengths in the interactions between N atom and C atom in the nitrile ligands shows discrepancies in the corresponding values (Figure 2). These discrepancies are large enough to suggest that the origin of the phenomenon should be bond-stretching isomerism. With this in mind EELS studies were carried out on both isomers.

### 2.1. EELS Results

EELS is an excellent complementary method to X-ray analysis since it allows the measurement of precise elemental composition, and information on the nature of the chemical bonds in the sample [25]. The interactions of fast electrons with the specimen result in excitations of electrons into unoccupied energy levels in the conduction band as well as collective excitations of valence electrons. When a spectrum is obtained by analyzing the energy lost by the incident electrons, the region up to an energy loss of ≈50 eV is dominated by collective excitations of valence electrons (plasmon) and by interband transitions. At higher energy losses, ionization edges occur due to excitation of core electrons into the conduction band, providing a method for studying the unoccupied conduction states in a solid.

These core-level processes are mostly sensitive to final states since the initial states have narrow energy widths. Besides the well defined ionization edges, there is a fine structure superposed on the edge and extending up to about 50 eV from the edge onset, which is associated with the density of unoccupied states in the conduction band, known as the Energy Loss Near Edge Structure (ELNES).

Generally, for a particular elemental ionization edge, the observed ELNES exhibits a structure that is specific to the arrangement, as well as the type of atoms within the first coordination shell. Since the inner-shell excitation process is highly localized on a particular atomic site, if two different coordinations of one element co-exist within the same structure, then the individual contributions of the different sites to the observed ELNES is a simple linear sum weighted by the respective site occupancies. Consequently, this technique has been used in the determination of sp^2^/sp^3^ bonded carbon ratios in diamond-like films [26].

Besides the Density of States (DOS) shape of the ELNES, any excess energy from the ionization energy can be thought as a wave emanating from the ionized atom. If this wave has only a few eV, it undergoes plural elastic scattering from the surrounding atoms, appearing as a broad signal some 20–40 eV above the edge onset. This is known as multiple scattering resonance (MSR), arising from a resonant scattering event involving the excited electron and a particular shell of atoms. The energies of these features above the edge onset have been shown to be proportional to 1/r^2^, where r is the bond length from the ionized atom.

**Figure 3:**
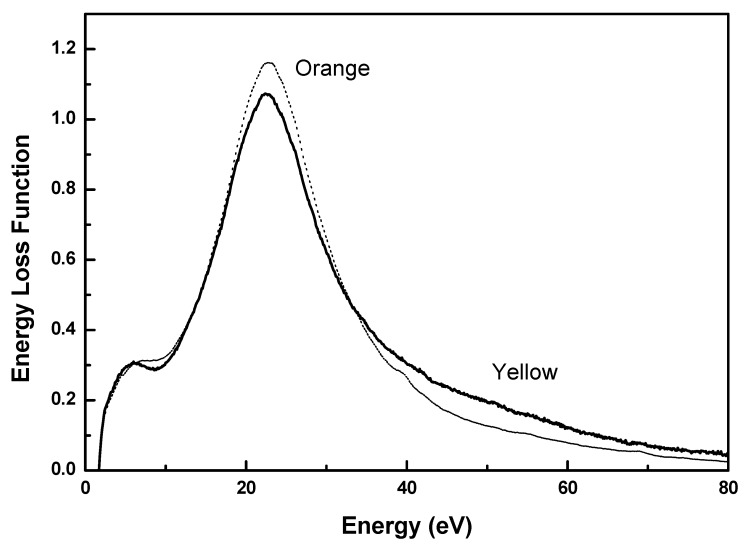
Energy loss function, Im(−1/ε), for yellow (continuous) and orange (dotted) samples, from the low energy loss region of EELS spectra.

Figure 3 shows the energy loss function for the yellow and orange samples. The dominant feature in the energy loss spectra is the volume plasmon, showing a well-defined maximum at 22.6 eV for both samples. The maximum in the energy loss function is the plasmon energy that can be written in a first approximation as:


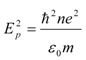


where, n is the total charge density, e the electron charge, m the mass of the electron and ε_0_ the permittivity of vacuum [27]. From Figure 3, we can assert that the electronic density is the same for both samples.

**Figure 4:**
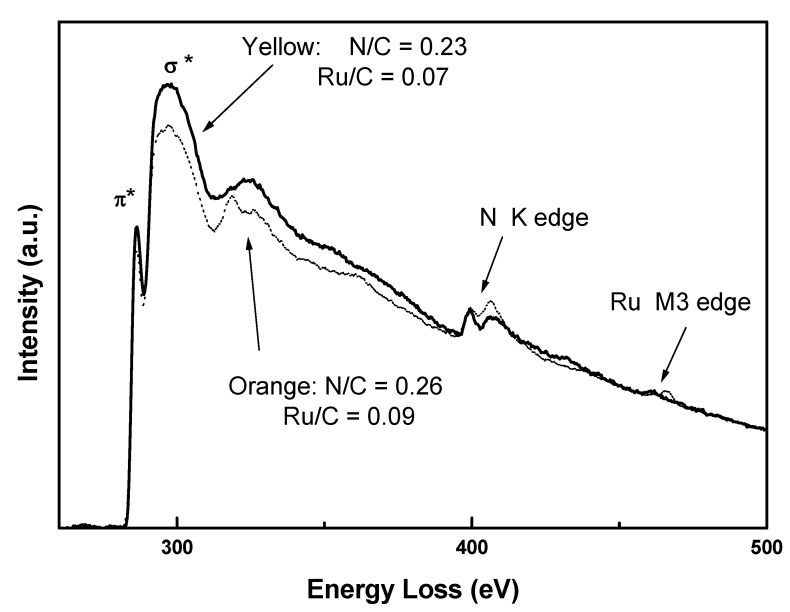
High-energy loss region of EELS spectra showing the K ionization edges from C and N and Ru M_3_ edge of yellow (continuous) and orange (dotted) samples.

Figure 4 shows energy-loss spectra in the high-energy region for both samples, where carbon (C) and nitrogen (N) K edge as well as the ruthenium (Ru) M_3_ ionization edge are clearly observed. Here the spectra have been normalized so that post edges above Ru ionization edge coincide. Spectra were background-subtracted and Fourier-ratio deconvoluted, to eliminate multiple scattering effects. Quantitative elemental analysis has been carried out by the usual Egerton method [27]. Elemental ratios obtained are: N/C = 0.23 ± 0.04 for yellow and N/C = 0.26 ± 0.04 for orange. Ru/C ratios are also very similar for both samples. Therefore, the difference in colour couldn’t be explained by variation in the composition.

**Figure 5:**
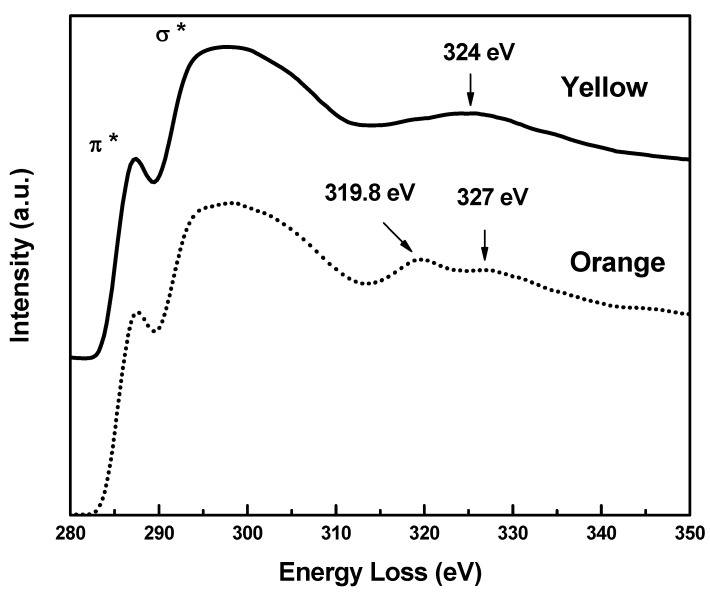
Carbon K Ionization edge, showing 1s-π* and 1s-σ* peaks. Signaled energies correspond to MSR peaks in yellow (continuous) and orange (dotted) samples.

Figure 5 shows energy-loss spectra for carbon K ionization edge for both samples, where spectra were shifted up for clarity. The ratios of sp^2^/sp^3^ obtained by fitting Lorentzian curves to π* and σ* peaks in the studied samples and the spectrum from pure graphite (not shown in the figure) are R_y_ = 83.7% and R_o_ = 95.1% respectively indicating a change in the hybridization of the isomers.

**Figure 6:**
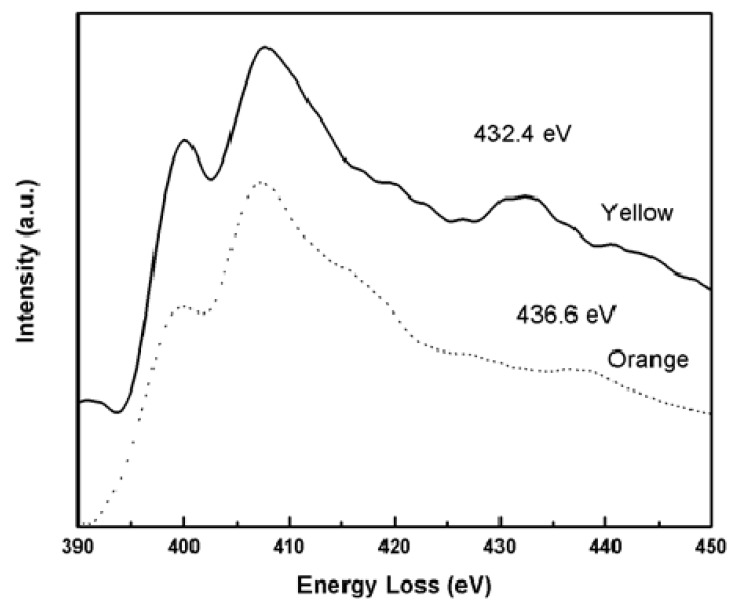
Nitrogen K ionization edge, showing π*, σ* and multiple scattering resonance signal in yellow (continuous) and orange (dotted) samples.

Figure 6 shows the energy-loss spectra for nitrogen K ionization edge for both samples, where spectra were displaced upwards for clarity. It is evident that the post edge fine structure has changed. The Multiple Scattering Resonance peak in the orange sample has moved to the right of the yellow sample, indicating that bond lengths in the orange isomer are smaller than in the yellow one.

We can make a semi-quantitative analysis on relative changes of bond-lengths in the yellow and orange samples. We can estimate the relative C–N bond-length variation between both samples by applying the empirical Equation [28]:


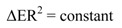
(1)

where, ΔE is the energy of the Multiple Scattering Resonant above the ionization threshold and R is the bond-length.

Equation (1) can be written, in our case, as:


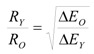
(2)

From Figure 5, we obtain the bond length in the yellow sample to bond length in the orange sample ratios for C atoms: R_Y_/R_O1_ = 1.04 and R_Y_/R_O2_ = 0.95, where R_O1_ and R_O2_ are related to the MSR peaks at 327 eV and 319.8 eV respectively. From the N post-edge structure in Figure 6 we have R_Y_/R_O_ = 1.04.

These results are qualitatively in good agreement with values obtained from X-ray analysis (see Figure 2). For C atoms, X-rays show that some bond lengths in yellow sample are increased with respect to the orange sample, as well as the opposite effect, e.g., in C12-C13 the calculated ratio is 0.990 and in C14-C15 the ratio is 1.014. On the other hand, for N atoms the bond lengths ratios are always greater than one (within the estimated uncertainty). In this case, the maximum change occurs in N2-C12 bond, the ratio being 1.03. Other minor bond stretching implies a minor shift in MSR peak positions and would be superposed on the main peak in EELS spectra.

### 2.2. Computational Results

Since our X-ray and EELS results point out the N2-C12 bond as that in which the maximum change occurred, we grossly estimated the interaction energy (E_int_) between the fragments A and B within the total molecule; the fragments A and B in both compounds, yellow and orange, were defined by the breaking of the N2-C12 bond (Figure 1 and Figure 2). To calculate the interaction energy in both cases, we carried out the single point energy calculations of each fragment (E_A_ and E_B_), and the single point energy calculations of the total molecule (E_t_). The E_int_ energy was obtained using the equation: E_int_ = E_t_− (E_A_ + E_B_).

Comparing the E_int_ energy calculated for the yellow and orange molecules, we obtained a difference between them in the order of van der Waals bond (3 kcal/mol). The optimized structure was achieved starting from the experimental structures of both the yellow and orange compounds, in both cases we reached the same optimized geometry. The relevant measurements for this species are shown in Figure 2. It is worth noting that there are important differences among the three structures, which will be discussed below. The important point is that these differences allow finding the source of the strange isomers.

Additionally, the TDDFT calculations performed in acetonitrile environment (see Table 1) are in good agreement with the experimental UV-Vis spectra. The experimental solution spectra were very similar: The same absorption maximums observed at λ = 240, 288 and 375 nm for both compounds and the only difference was noted in values of the extinction coefficients. The extinction coefficient of longer wavelength absorption of the orange compound was slightly (about 5–13% depending on the solvent) higher than that of the yellow sample. Interestingly that this difference in MeCN solution spectra was less than in the spectra run in other solutions such as CH_2_Cl_2_ and MeOH [24].

**Table 1:** TDDFT exited states spectra performed in MeNC environment. Main contribution (MC) to each exited state with the HOMO corresponding to the 92 orbital. Excitation energies (EE, eV), wave lengths (λ, nm), percentage of probability (%P) that the transition occurs and oscillator strengths (f) computed for both yellow and orange isomers.

Yellow compound					Orange compound				
MC	%P	EE	λ	f	MC	%P	EE	λ	f
S92→93	69.29	2.56	484	0.0016	S92→93	69.04	2.57	482	0.0028
S91→93	70.32	2.67	464	0.0002	S91→93	70.28	2.68	463	0.0003
S92→94	65.77	2.96	418	0.0023	S92→94	65.34	2.99	415	0.0021
S91→94	70.44	3.07	404	0.0002	S91→94	70.23	3.10	400	0.0005
S90→93	60.34	3.21	387	0.1327	S90→93	59.56	3.19	389	0.1140
S90→94	65.96	3.40	365	0.0219	S90→94	65.36	3.45	359	0.0362
S89→93	59.50	3.90	318	0.0678	S89→93	53.64	3.99	311	0.0690
S92→95	62.05	3.94	315	0.0013	S92→95	51.16	4.01	309	0.0166
S92→96	48.26	4.08	304	0.0227	S92→96	52.78	4.12	301	0.0327
S88→93	53.81	4.09	303	0.0104	S88→93	62.60	4.13	300	0.0037

**Figure 7:**
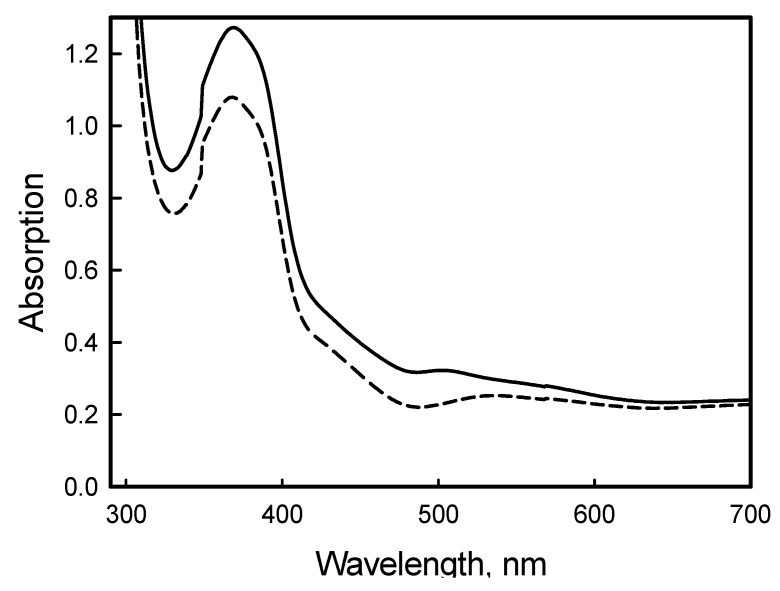
UV-vis spectra of MeCN solutions of yellow (dashed line) and orange (solid line) compounds.

However it was also noted a little more pronounced kind of shoulder around 480–500 nm for the orange sample, but the difference was very difficult to detect numerically. This part of the UV-Vis spectra run in MeCN solution for the yellow and orange compounds is shown in Figure 7 for clarity.

Explicitly, for both the yellow and orange compounds, the TDDFT calculations predict that the vertical transition more strongly absorbed with similar intensities and probabilities (f = 0.1327, %P = 60.26; and f = 0.1140, %P = 59.45 respectively) corresponds to the transition HOMO-2 → LUMO (3.21 and 3.19 eV) localized at 387 and 389 nm respectively. Comparing with the UV-Vis spectra, these theoretical transitions are matching with an experimental wide band observed for both compounds at 374 nm. This absorption band is at near ultra-violet range and does not impact much in colour; an absorption in longer wavelength should be responsible for the colour differences. Our TDDFT calculations show other very similar absorptions at 400–404 and 415–418 nm for both compounds (transitions HOMO-1 → LUMO+1 and HOMO → LUMO+1). However the biggest difference is observed for the HOMO → LUMO vertical transition localized at 480 nm (484 nm for the yellow and 482 nm for the orange). It has approximately the same probability for both compounds (69.29% and 69.04% respectively), but is ~42% more strongly absorbed by the orange isomer. This coincides with the experimental observation of more pronounced shoulder in this region for the orange sample (see Figure 7 above).

**Figure 8:**
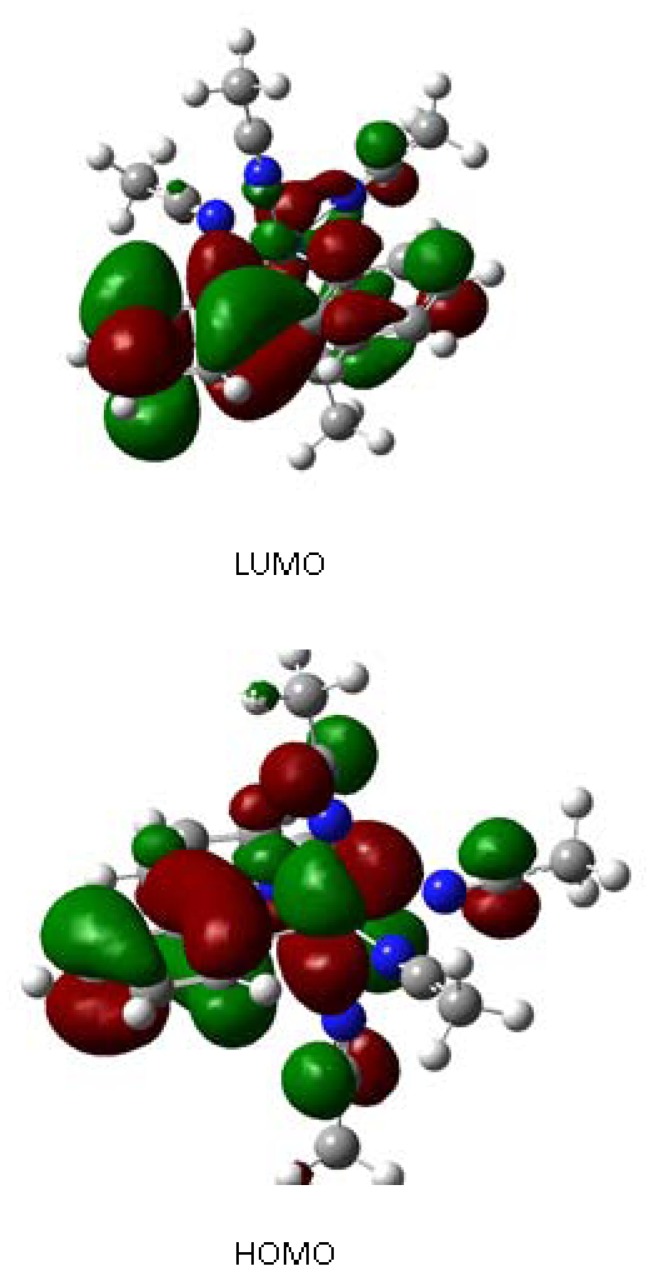
HOMO, LUMO set for yellow isomer.

Nevertheless, no dissimilarities were found when we examined the corresponding HOMO-1, HOMO, LUMO and LUMO+1 of both structures (Figure 8). At this point the most important question is what is the source of these changes. A qualitative analysis of the wave functions may suggest an answer. It seems that the phenomenon occurs due to the presence of the phenyl-pyridine ligand (3), its *trans* effect and the nature of its frontier orbitals. The HOMO-LUMO gap of free 3 is relatively large (5.7 eV) but the nature of the LUMO is an antibonding π orbital. However it is placed so far from the HOMO that a strong interaction between the frontier orbitals is not expected.

This situation changes dramatically when the fragment 3 forms part of the organometallic compounds, in these cases the HOMO-LUMO gap for both complexes are 2.456 eV for the orange compound and 2.479 eV for the yellow compound. Now the gap is narrow in both cases and it is expected some kind of interaction of both orbitals even more considering that the LUMO is an antibonding molecular orbital very appropriated for backbonding (see Figure 8).

Thus, the difference between the gaps is only 0.023 eV; therefore in an idealized situation the compound would present two local minima corresponding to the structures, which impose different effect on the acetonitriles in the *trans*-position with respect to the phpy ligand. Indeed, the N2-C12 bond length, that belongs to the fragment of acetonitrile in the position *trans* to the nitrogen atom of phpy, is 1.117 Å for the orange compound; whereas the same bond in the yellow sample is 1.137 Å due to the difference in energy caused by the variation in the backbonding. This difference is not enough to describe these two forms as bond stretch isomers, but it may suggest an exchange hybridization case. This can be verified by analyzing the dihedral angles between Ru-N2-C12-C13 (Figure 2) which have the values of 22.67° for the orange and 25.0° for the yellow compound. This assumption is in agreement with the EELS result that the difference between both compounds is the sp2/sp3 ratio. The transition between both different hybridization structures was computed, but no definite transition state was found. The failure to find a double minimum on the potential energy curve has been reported for various transition metal complexes searched for bond stretch isomerism [12,18]. Some authors proposed existence of fluxionallity between both structures in solution, which can be trapped by crystallization. However the hypothesis of simple thermo-fluctuations in solution can’t be a plausible explanation for the phenomenon of the yellow and orange Ru(II) compounds. The transformation of the yellow form into the orange one or *vice versa* has never occurred in any kind of solvent, even at elevated temperatures. Thus, absence of the definite transition between these two forms demonstrated by the modelling is confirmed by the experimental observations. The yellow compound converts into the orange form only in the presence of pyridine or bipyridine molecules. These molecules in an attempt of ligand substitution reaction somehow cause the fluctuation in relatively labile acetonitrile ligands that results in the colour change. Thus it may be a case of “induced” fluxionality.

## 3. Experimental

### 3.1. Synthesis of the Ruthenium(II) Complexes

The yellow and orange forms of the ruthenium complex, [Ru(*o*-C_6_H_4_-py)(MeCN)_4_]^+^, were synthesized as described in reference [24]. Their UV-Vis spectra were obtained in freshly prepared MeCN solutions using a Varian Cary 400 UV-Vis spectrophotometer.

### 3.2. EELS Spectroscopy

Electron energy loss spectra were obtained using a Gatan Parallel Electron Energy Loss Spectrometer (PEELS model 766) attached to a Philips CM-200 transmission electron microscope (TEM). Spectra were taken in diffraction mode with 0.3 eV/ch dispersion, an aperture of 3 mm and a collection semi-angle of 2.8 mrad. The resolution of the spectra was determined by measuring the full width at half-maximum (FWHM) of the zero-loss peak. This was typically close to 1.5 eV for the low energy region, when the TEM was operated at 200 kV. The EELS spectra were corrected for dark current and readout noise. The channel-to-channel gain variation was minimized normalizing the experimental spectrum with the gain spectrum of the spectrometer obtained independently. Spectra in the high-energy region were background-subtracted fitting the pre-edge backgrounds with a power-law function and then Fourier-Ratio deconvoluted to remove multiple scattering components from the spectra. In the low-energy region, spectra were Fourier-Log deconvoluted to obtain single scattering distributions S(E).

### 3.3. Molecular Modelling Details

All calculations were carried out using the BPW91 scheme [29,30] with the 6-31G* basis, the code used was Gaussian03 [31] on single point calculation performed on the published x-ray structures. TDDFT calculations were carried out at the same level of theory in order to simulate the visible spectra previously reported. The application of TDDFT in the field of quantum chemistry is relatively recent [32,33], and a number of problems have been identified in its implementation. Although the exact functional E_xc_ of DFT is not known, there are a number of approximate forms that give useful results. In the case of TDDFT the situation is more complicated due to the required functional A_xc _is time dependent on the electron density. The simplest approximation is the so called, Adiabatic Approximation, which uses the zero frequency limit of A_xc_ for treatments of finite frequency perturbations. This is justified for the low-frequency domain. In the high frequency domain some overestimation of the excitation energies is observed and certain excitations out of the σ-system are underestimated. In general, better results are obtained for functional that provide an exchange-correlation potential with correct asymptotic behaviour (−1/r) [34,35].

## 4. Conclusions

The phenomenon of the formation of two forms distinguished by colour (yellow and dark orange) in the cyclometalated Ru(II) complex, [Ru(o-C6H4-py)(MeCN)4]+, was studied by means of EELS spectroscopy and DFT calculations. EELS spectroscopy confirmed the same composition of both forms but revealed a small difference in the hybridization states of the carbon atoms and variations in the C–C and C–N bond lengths of the ligands. Careful analysis of X-ray data also showed minute differences in some bond length of the acetonitrile ligands. The calculated energy surfaces for these two forms demonstrated the existence of only one minimum and thus the yellow and orange compounds could not be considered as a case of bond-stretch isomerism. However a small difference in energy resulting from backbonding between the yellow and orange forms was found. This affects the geometry of the compounds and leads to a change in the hybridization of the carbon in the *trans*- acetonitrile ligand. The observed difference in colour is rather caused by slight difference in the hybridization. Thus, the results of theoretical modelling coincide well with the experimental observations.
